# Functional Assessment of Hypertrophic Phenotype Cardiomyopathies Using Combined Cardiopulmonary Exercise Testing and Echocardiography: A Pilot Single-Centre Study †

**DOI:** 10.3390/jcm15093470

**Published:** 2026-05-01

**Authors:** Mattia Scolari, Iacopo Fabiani, Lorenzo Bazan, Giancarlo Todiere, Chiara Arzilli, Christina Petersen, Ignazio Alessio Gueli, Eleonora Benelli, Carmen Corciulo, Claudio Passino

**Affiliations:** 1 Health Science Interdisciplinary Center, Sant’Anna School of Advanced Studies, 56127 Pisa, Italy; 2 Fondazione Toscana Gabriele Monasterio (FTGM), 56124 Pisa, Italy; 3 Division of Cardiology, University of Pisa, 56126 Pisa, Italy

**Keywords:** cardiopulmonary exercise testing, CPET-ESE, exercise stress echocardiography, heart failure with preserved ejection fraction, hypertrophic cardiomyopathy, left ventricular hypertrophy, peak VO_2_, transthyretin cardiac amyloidosis

## Abstract

**Background**: In patients with left ventricular hypertrophy, resting structural parameters alone may not explain exertional symptoms. Hence, we investigate whether combined Cardiopulmonary Exercise Testing- Exercise Stress Echocardiography (CPET-ESE) can provide an integrated functional characterisation of hypertrophic phenotypes. **Methods**: As a preliminary investigation, this prospective single-centre pilot study enrols 43 patients, categorised into: obstructive hypertrophic cardiomyopathy (*n* = 19), transthyretin cardiac amyloidosis (*n* = 15), or preserved-ejection-fraction hypertrophic phenotypes (*n* = 9). Patients undergo symptom-limited semi-supine CPET-ESE on an electronically braked cycle ergometer with an individualised ramp protocol. Peak effort is defined by symptom limitation and respiratory exchange ratio criteria (RER≥1.05), while peak VO_2_ is defined as the highest 30 s averaged value. **Results**: Exercise responses differ across phenotypes. Patients with obstructive hypertrophic cardiomyopathy have higher peak VO_2_ than the other groups, despite their lower chronotropic reserve. The preserved-ejection-fraction hypertrophic group shows lower peripheral oxygen extraction, whereas transthyretin amyloidosis shows a mixed central and peripheral limitation pattern. Right ventricle–pulmonary artery uncoupling is observed in the latter two groups. **Conclusions**: The use of CPET-ESE may help describe distinct physiological exercise profiles in hypertrophic phenotypes, but these findings should be considered exploratory. The small, heterogeneous and single-centre cohort precludes definitive mechanistic or predictive conclusions and supports the need for larger validation studies.

## 1. Introduction

Left ventricular hypertrophy (LVH) represents an increase in myocardial mass due to wall thickening, chamber enlargement, or both [1]. According to ASE/EACVI criteria, LVH is defined by a left ventricular mass index (LVMi) $>115\;g/m^{2}$ in men and >95 $g/m^{2}$ in women [2]. As demonstrated by the Framingham Heart Study, LVH is a strong, independent predictor of cardiovascular morbidity and mortality [3].

The presence of myocardial thickening is a hallmark feature shared by various cardiac conditions, including two particularly important causes:

- Hypertensive heart disease and Heart Failure with Preserved Ejection Fraction (HFpEF) phenotype. Chronic pressure overload induces myocyte hypertrophy, interstitial fibrosis, and diastolic dysfunction, progressing to HFpEF [1]. Echocardiography typically shows concentric LVH, preserved ejection fraction (EF), mild diastolic impairment, and left atrial enlargement [4].
- Hypertrophic cardiomyopathy (HCM), a genetic disorder mainly linked to autosomal dominant mutations of sarcomeric proteins (*MYH7*, *MYBPC3*) [5]. It is characterised by asymmetric septal hypertrophy and frequently features dynamic left ventricular outflow tract obstruction (LVOTO) driven by systolic anterior movement (SAM) of the mitral valve [6,7].

However, not all imaging-defined LVH reflects a true increase in myocardial mass due to cardiomyocyte hypertrophy. In a subset of conditions, apparent wall thickening results from extracellular infiltration, a phenomenon referred to as pseudo-hypertrophy [8].

Cardiac amyloidosis represents the prototypical model of pseudo-hypertrophy. It is an infiltrative cardiomyopathy caused by extracellular deposition of misfolded amyloid fibrils, leading to concentric wall thickening, HFpEF, reduced stroke volume (SV), and the characteristic discordance between increased wall thickness and low QRS voltages on ECG [9].

Notably, the severity of LVH-related symptoms often correlates poorly with the absolute degree of myocardial thickening [10,11]. This clinical–structural discrepancy suggests that resting morphological assessments are insufficient to explain the complex pathophysiology of the disease, highlighting the need for a transition toward comprehensive functional evaluation [4,7].

**Figure 1:**
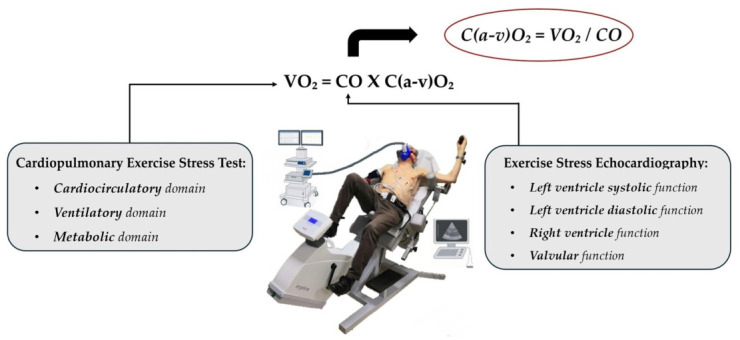
Combined CardiopulmonaryExercise Testing-Exercise Stress Echocardiography (CPET-ESE) methodology. This setup utilizes a semi-supine electronically braked cycle ergometer with an individualised ramp protocol designed for an approximately 12 min exercise phase. While the CPET component offers an integrated evaluation of the cardiovascular, ventilatory, and metabolic systems through gas exchange analysis, simultaneous ESE allows for a targeted focus on cardiac functional and structural changes under physical stress, providing a comprehensive overview of the patient’s physiological response. Adapted and modified from [14].

The latest methodological advance to assess exercise intolerance is the simultaneous integration of Cardiopulmonary Exercise Testing with Exercise Stress Echocardiography (CPET-ESE), represented in Figure 1. In this approach, echocardiographic images are acquired during exercise at predefined, physiologically relevant stages, while gas exchange and ventilatory parameters are continuously monitored [12].

This simultaneity enables direct, time-matched correlation between metabolic demand, ventilatory response, and dynamic cardiovascular performance, allowing a comprehensive mechanistic assessment of exercise limitation [13]. Although CPET-ESE is not yet included in formal guideline recommendations and lacks a universally standardised protocol, its clinical use is steadily increasing, supported by evidence of reproducibility and incremental value for phenotypic characterisation, prognosis, and personalised therapeutic decision-making [14].

Such an integrated assessment may prove particularly effective for differentiating complex heart failure phenotypes and defining disease stages [15,16]. Specifically, it could potentially facilitate the clinical distinction between HFpEF and heart failure with reduced ejection fraction (HFrEF) while also aiding in the possible identification of early versus overt HFpEF [17].

Pathophysiological insights have also emerged in HCM, where exercise intolerance may be driven by a complex interplay between diastolic dysfunction and LVOTO [18]. By stratifying patients based on the presence of obstruction—whether resting, latent, or absent—and the degree of diastolic impairment, a study suggested that functional capacity may be most severely compromised when both conditions coexist at rest [19].

Regarding cardiac amyloidosis, evidence supporting the clinical application of CPET-ESE remains more limited. However, data comparing patisiran-treated patients with untreated cohorts provide early insights. Specifically, untreated individuals appear to show a more pronounced decline in inotropic reserve and a worsening VE/VCO_2_ slope [20].

## 2. Aims

This prospective and observational study hypothesizes that different hypertrophic phenotypes—such as hypertrophic cardiomyopathy with obstruction (HCMO), HFpEF, and transthyretin amyloid cardiomyopathy (ATTR-CA)—exhibit distinct “exercise signatures.” These signatures likely reflect differing limitations in cardiac output reserve, pulmonary vascular response, and/or systemic oxygen extraction capacity. By identifying these phenotypic profiles through CPET-ESE, clinicians may gain more accurate diagnostic and prognostic tools and better target therapies in a patient-specific manner. The primary aim is to validate CPET-ESE as an integrated tool for the functional characterisation of hypertrophic phenotypes and for identifying the distinct pathophysiological mechanisms underlying exercise limitation. The secondary objectives are to identify independent predictors of functional impairment—such as peak VO_2_, ventilatory efficiency (VE/VCO_2_ slope), and peak [C(a-v)O_2_]—using multivariable regression models that incorporate clinical variables, circulating biomarkers, resting echocardiographic parameters, and variables derived from ESE alone, without the direct inclusion of CPET measurements.

## 3. Materials and Methods

### 3.1. Study Population

Given the heterogeneous nature of LVH, a structured etiological classification is adopted to allow pathophysiologically coherent comparisons across groups. Within this methodological framework, the differential diagnosis between physiological LV remodeling (athlete’s heart) and pathological hypertrophy is beyond the scope of the present investigation. Accordingly, the study population exclusively includes patients with an established clinical diagnosis of pathological hypertrophic phenotypes based on current guideline-supported criteria. In line with this rationale, enrolled patients are categorised into three predefined LVH phenotypes:

- HFpEF, including hypertensive heart disease and non-obstructive HCM, diagnosed using ESC criteria: LV wall thickness ≥12mm, HF symptoms and signs, EF ≥50%, and evidence of diastolic dysfunction or elevated natriuretic peptides levels [21]. Furthermore, non-obstructive HCM is defined by the absence of significant LVOTO, corresponding to an LVOT gradient ≤30mmHg both at rest and during exercise [7];
- ATTR-CA, confirmed by bone scintigraphy (Perugini score 2–3) and absence of monoclonal protein, or alternatively by endomyocardial biopsy with mass spectrometry in ambiguous cases [22];
- HCMO, defined by a maximum LV wall thickness ≥15mm, preserved EF, and the presence of LVOTO, defined as an LVOT gradient >30mmHg at rest, or induced by Valsalva maneuver, or detected during exercise [7].

Exclusion criteria include:

- Reduced EF (<50%);
- Arrhythmias on ECG screening or prolonged QTc;
- Syncope or ventricular arrhythmia within the 6 months prior to stress testing;
- Severe valvular heart disease:
  – Severe aortic stenosis: mean transvalvular gradient ≥40mmHg and/or aortic valve area $\leq 1.0\;{\mathrm{cm}}^{2}$;
  – Severe MR: regurgitant volume ≥60mL/beat or effective regurgitant orifice area $\geq 0.40\;{\mathrm{cm}}^{2}$;
  Valvular lesions below these thresholds (e.g., a calcified aortic valve with a mean gradient of 30 mmHg) are classified as moderate and are therefore not considered exclusion criteria;
- History of previous cardiac surgery;
- Established haematological conditions linked to anemia or significant blood abnormalities, including anemia (Hb <12g/dL for women; <13 g/dL for men);
- Severe Chronic Obstructive Pulmonary Disease (COPD), particularly in the presence of severe functional impairment, defined as markedly reduced exercise tolerance, significant dyspnea on minimal exertion (modified Medical Research Council scale, mMRC ≥3), forced expiratory volume in 1 s (FEV1) <50% predicted, or oxygen desaturation during physical activity.

### 3.2. Study Procedures

**Figure 2:**
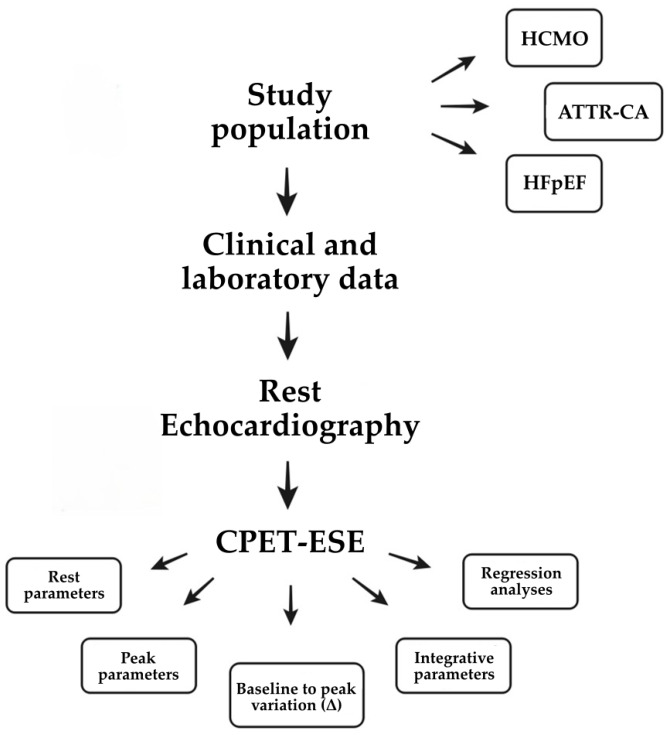
Flowchart of the study protocol. Clinical and laboratory data are first collected, followed by comprehensive resting echocardiography to characterise cardiac structure and function. Participants then undergo CPET-ESE, which yields multiple categories of physiological information: resting exercise parameters, peak exercise responses, baseline-to-peak (Δ) variations, and additional integrated cardiometabolic indices. These datasets are subsequently used for comparative analyses across phenotypes (HCMO, ATTR-CA, HFpEF) and for regression modelling.

To ensure a comprehensive and standardised analysis, the study protocol is articulated into three sequential phases, as reported in Figure 2, progressing from clinical profiling to structural evaluation and, finally, to integrated functional assessment.

1. A detailed evaluation of baseline characteristics and cardiovascular profile was planned together with an assessment of key biomarkers including NT-proBNP, high-sensitivity troponin, and estimated glomerular filtration rate (eGFR);
2. Resting transthoracic echocardiography is performed in accordance with ASE/EACVI recommendations, with quantification of structural and functional parameters relevant to LVH phenotyping [2];
3. Combined CPET-ESE, described in detail in the following subsections.

### 3.3. Integrated CPET-ESE Protocol

**Figure 3:**
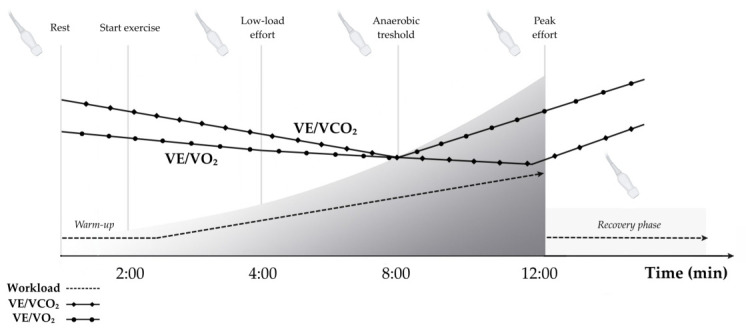
Illustration of the CPET-ESE protocol. The exercise phase is designed to last approximately 12 min, using an individualised ramp to ensure a progressive and controlled increase in workload after a 2 min warm-up period. Gas-exchange variables rise or fall in a characteristic pattern: VE/VCO_2_ and VE/VO_2_ are continuously monitored, and their trajectories assist in identifying the anaerobic threshold (AT). Echocardiographic imaging is acquired at predefined stages—rest, low-load effort, AT, peak exercise, and immediately post-exercise. The figure was adapted and modified with permission from [13].

The combined CPET-ESE approach captures the dynamic interactions between cardiac, ventilatory and metabolic function during graded exercise. A detailed overview of the assessed variables is provided in Table 1 and further expanded in the Supplementary Materials. The full CPET-ESE protocol entails a continuous, multi-stage evaluation ranging from rest stage to post-exercise recovery, as illustrated in Figure 3. For this preliminary analysis, however, we focus exclusively on absolute values at baseline and peak exercise, alongside the dynamic Δ responses.

#### 3.3.1. CPET Methodology

Patients undergo a symptom-limited, semi-supine CPET on an electronically braked ergometer, with clinical status monitored via continuous 12-lead ECG, SpO_2_, and automated blood pressure measurements throughout the procedure. To ensure an optimal exercise duration of about 12 min, an individualised ramp protocol is employed, after a brief warm-up phase. Gas exchange is analysed on a breath-by-breath basis, measuring oxygen uptake (VO_2_), carbon dioxide production (VCO_2_), and minute ventilation (VE), with predicted VO_2_ values derived from Wasserman equations.

The anaerobic threshold (AT) is determined using a multi-method approach, integrating the V-slope method, the nadir of the ventilatory equivalent for oxygen (VE/VO_2_) prior to a concomitant rise in VE/VCO_2_, and end-tidal gas trends (an increase in PETO_2_ with stable PETCO_2_) [23] Under standard conditions, a test is validated as reaching peak effort when the respiratory exchange ratio (RER) is ≥1.05. However, in cases where this threshold is not met, peak effort is defined by symptom limitation. Consequently, Peak VO_2_ is defined as the highest 30 s rolling average achieved during the final stage of the exercise protocol. Findings are visually cross-referenced and confirmed by two independent observers.

#### 3.3.2. ESE Methodology

Echocardiographic acquisition is coupled with gas exchange analysis to correlate cardiac haemodynamic changes directly with metabolic workload. Measurements are performed at five key intervals:

- Resting baseline;
- Low workload within the first 4 min and a heart rate (HR) <100 bpm;
- Anaerobic threshold (AT), marked by a RER ≈1.0;
- Peak exercise, with RER ≥1.05 or symptom-limitation;
- Early recovery phase, within the first 2–3 min post maximal exercise.

At each stage, a comprehensive assessment of cardiac geometry and function is performed. This includes ventricular performance, diastolic indices, and valvular dynamics, as detailed in Table 1 and in Supplementary Materials. To ensure data robustness, all echocardiographic parameters are averaged over three consecutive cardiac cycles, or a 5 s interval for patients in atrial fibrillation.

### 3.4. Statistical Analysis

Comparative analysis across phenotypes is performed using parametric or non-parametric tests based on data distribution. Continuous variables are examined with one-way ANOVA. Categorical variables are assessed using the $\chi^{2}$ test. Statistical significance is set at p<0.05 for all analyses. To investigate the determinants of functional capacity, multivariable modelling is subsequently applied. Predictors of peak VO_2_, ventilatory efficiency (VE/VCO_2_ slope), and peak C(a-v)O_2_ are identified through multivariate linear regression, including only candidate predictors demonstrating a p<0.10 at univariate screening. Regression coefficients (β) are used to quantify the strength and direction of associations, while multicollinearity among predictors is assessed using the variance inflation factor (VIF). Model performance and discriminatory ability are evaluated using adjusted R^2^; to quantify explained variance, receiver-operating characteristic (ROC) curves and area under the curve (AUC) for classification tasks, and Bland–Altman analysis to assess agreement between measured and predicted values. All analyses are conducted using complete-case analysis, as no significant missing data were present.

## 4. Results

### 4.1. Clinical Parameters and Biomarkers

The study population includes 43 patients with a mean age of 68±10 years, who were predominantly male (84%, n=36); females accounted for 16% (n=7). Most patients were in New York Heart Association functional classification (NYHA) class II (74%, n=32), whereas the remaining 26% were in class I (n=11). Notably, despite a significantly younger mean age (62±9 years, p=0.001), HCMO patients showed the highest proportion of subjects in NYHA class II (95%, n=18, p=0.024).

Regarding biomarkers, Log-transformed cardiac Troponin T (logTnT) presents a median value of 1.261 (IQR 1.055–1.433) in the overall population, with significantly higher levels observed in ATTR-CA compared with the other groups (median 1.582, IQR 1.359–1.655, p<0.001). NT-proBNP shows a median value of 933 pg/mL (IQR 459–1573). Renal function is preserved overall, with creatinine levels of 1.02±0.23 mg/dL and an eGFR of 75±17 mL/min/1.73 m^2^, without significant differences among groups.

The most frequent comorbidities are hypertension (79%, n=34) and dyslipidemia (65%, n=28), followed by AF (35%, n=15), Ischemic Heart Disease (23%, n=10) and Diabetes Mellitus (12%, n=5). Overall, 40% of patients (n=17) are current or former smokers. Three subjects (7%) have an implanted pacemaker.

Among pharmacological therapies, β-blockers are the most prevalent prescribed (72%, n=31), with the highest usage observed in the HCMO subgroup, despite not reaching statistical significance (p=0.061). Further subgroup details are reported in Table 2.

### 4.2. Baseline Echocardiography

Baseline echocardiographic parameters are summarised in Table 3. The overall study population exhibit increased LVMi (158±38 g/m^2^) together with marked left ventricular wall thickening, reflected by an interventricular septal thickness in diastole (IVSd) of 17.0±2.9 mm and a posterior wall thickness in diastole (PWd) of 13.1±2.4 mm. These findings are consistent with a hypertrophic remodeling pattern shared across the three groups. Notably, IVSd is significantly greater in the HCMO group (18.2±3.3 mm, p=0.025).

In the general population, EF is 59±6%, with all groups showing preserved EF, although values are slightly higher in HCMO (61±5%; p=0.022). Left ventricular cavity dimensions remain within normal limits, with a left ventricular end-diastolic diameter (LVEDD) of 48±5 mm and a left ventricular end-diastolic volume (LVEDV) of 121±33 mL.

The diastolic profile shows impaired relaxation, with a mean E/e′ ratio of 13.7±4.7, associated with left atrial enlargement (LAVI 40.4±9.6 mL/m^2^), suggesting chronically elevated filling pressures. Among the groups, HFpEF shows significantly lower E/e′ values compared with the others (10.11±3.06, p=0.030).

Right ventricular function is preserved, with a TAPSE of 21±4 mm and a PAPs of 34±9 mmHg. Regarding valvular function, significant MR is present in 19% of patients (n=8), with significantly more prevalence in the HCMO group (*n* =6.32%, p=0.024), while significant tricuspid regurgitation is observed in 7% of patients (n=3).

### 4.3. CPET-ESE

A summary of CPET-ESE parameters is provided in Table 4. At rest, in the overall cohort, SBP (131±19 mmHg), DBP (74±10 mmHg) and HR (64±12 bpm) are within normal limits, with a mean double product (DP) of 8,297 ± 2,253, reporting no significant differences among groups.

The systolic function is preserved (SVi 37±9 mL/m^2^; CI 2.3±0.6 L/min/m^2^), and diastolic function shows a mean E/e′ of 12.7±5.0. Right ventricular function is preserved, with a mean TAPSE of 22±3 mm and basal PASP of 32±8 mmHg, showing no significant differences among groups.

Resting metabolic function is reduced (VO_2_ 3.85±1.57 mL/min/kg), with slightly increased peripheral oxygen extraction (C(a-v)O_2_ 7.2±3.1 mL/100 mL). Notably, HCMO patients exhibit significantly higher resting metabolic activity (basal VO_2_ 4.92±1.40 mL/min/kg), p<0.001; basal C(a-v)O_2_ 8.88±2.64 mL/100 mL, p<0.001), as compared to the other groups. Patients with ATTR-CA show the lowest basal VO_2_ (3.04±1.36 mL/min/kg), while the HFpEF group reports the lowest basal C(a-v)O_2_ (5.50±1.36 mL/100 mL).

At peak exercise, the hemodynamic response is appropriate in the general population, with increases in SBP (184±27 mmHg), DBP (91±15 mmHg), and mean HR (106±19 bpm), resulting in a doubling of the double product (19,892±5551).

Mean values for SVi and CI reach 48±12 mL/m^2^ and 5.1±1.4 L/min/m^2^, respectively, showing no significant variation across the three cohorts. By contrast, variations in diastolic function are observed: HFpEF shows lower filling pressures (peak E/e′ 8.7±3.8) compared with both ATTR-CA (12.3±4.0) and HCMO (13.4±4.8), with a significant overall difference (p=0.033). The HCMO group also demonstrates a higher prevalence of significant MR at peak exercise (63%, n=12; p=0.003) compared with ATTR-CA (3/15) and HFpEF (0/9).

Right ventricular contractile function is generally preserved, with TAPSE at peak reaching 27±5 mm. A peak PASP of 45±18 mmHg is reached, with no significant differences between groups. Notably, ATTR-CA and HFpEF show decoupling of TAPSE/PASP during effort (ΔTAPSE/PASP: ATTR-CA −0.18±0.16; HFpEF −0.10±0.16; HCMO 0.10±0.40; p=0.045).

Peak VO_2_ results 14.92±3.98 mL/min/kg in the general population, supported by an adequate increase in C(a-v)O_2_ (12.77±3.37 mL/dL). Again, the HCMO group shows the highest values in both parameters, with a peak VO_2_ of 16.74±2.84 mL/min/kg (p=0.020) and a peak C(a-v)O_2_ of 14.88±2.94 mL/100 mL (p<0.001).

In this assessment, the HFpEF group reports both the lowest VO_2_ (13.27±3.34 mL/min/kg) and C(a-v)O_2_ (10.46±1.47 mL/100 mL), with values comparable to ATTR-CA. Furthermore, this result is supported by the proportion of patients reaching AT, which is markedly higher in HCMO (17/19, 90%; p<0.001) compared to ATTR-CA (1/15, 7%) and HFpEF (2/9, 22%).

Despite these observations, HCMO shows the lowest chronotropic reserve values (39±12%; p=0.009), with almost all patients (n=18, 95%; p<0.001) failing to reach the 62% cut-off threshold for chronotropic incompetence.

In the general population, the VE/VCO_2_ slope is 28.3±5.1 and does not differ significantly among groups, whereas the CO/VO_2_ slope is at its highest in HFpEF (7.25±3.02; p=0.009).

### 4.4. Regression Analyses

Regression analyses are performed to identify independent predictors of peak VO_2_, VE/VCO_2_ slope, and peak C(a-v)O_2_ in the overall cohort.

In the multivariable regression, peak VO_2_ is independently predicted by baseline TAPSE (β=+0.40; VIF 1.06), peak CI (β=+0.40; VIF 1.06), and age (β=−0.37; VIF 1.04). This model explains a substantial proportion of the variance in the cohort (adjusted $R^{2}=0.57$; p<0.001).

Regarding the VE/VCO_2_ slope, peak CI (β=−0.33; VIF 1.08), age (β=0.22; VIF 1.14), and LAVI (β=+0.41; VIF 1.22) emerge as independent predictors, accounting for significant variability (adjusted $R^{2}=0.41$; p<0.001).

Finally, stepwise regression identifies peak CI (β=−0.49; VIF 1.07), age (β=−0.31; VIF 1.23), baseline MR (β=+0.35; VIF 1.22), and log NT-proBNP (β=−0.50; VIF 1.39) as independent predictors of peak C(a-v)O_2_, with an adjusted $R^{2}=0.58$ (p<0.001).

## 5. Discussion

### 5.1. Baseline Phenotyping

Already at baseline, both clinical, laboratory and echocardiographic data allow identification of differences among the three groups. The mean age is higher in patients with both ATTR-CA and HFpEF compared with those with HCMO.

The burden of comorbidities follows a similar gradient, being greater in the older groups. In particular, comorbidities are not only more prevalent but also more heterogeneous in the ATTR-CA group, consistent with the systemic nature of the disease [24].

Serum biomarkers show significantly higher troponin levels in patients with amyloidosis compared with HFpEF and HCMO, reflecting greater myocardial injury.

Notably, despite their younger age and more favorable biomarker profile, patients with HCMO show a higher proportion of symptomatic individuals in NYHA class II, likely explained by the prominent role of LVOTO [25].

Baseline echocardiographic findings support this interpretation. In the HCMO group, septal hypertrophy is more pronounced and accompanied by higher aortic outflow velocities. In addition, moderate-to-severe MR is more frequently observed in HCMO, a finding related to SAM.

Regarding therapy, although not significant, β-blockers are more frequently prescribed in HCMO compared with ATTR-CA and HFpEF, consistent with the need to modulate HR in order to reduce LVOTO.

In contrast, patients with ATTR-CA and HFpEF exhibit increased wall thickness with more symmetric distribution.

### 5.2. CPET-ESE Phenotyping

Although these preliminary findings should be interpreted with caution, differences between the groups can be observed. Regarding basal data, despite similar rest CI and SVi across groups, HCMO patients show higher rest VO_2_ and rest C(a-v)O_2_, likely reflecting greater skeletal muscle mass and, in the context of LVOTO and more pronounced diastolic dysfunction, increased reliance on peripheral oxygen extraction.

Peak exercise data provide further potential insight into group-specific metabolic and hemodynamic responses. Patients in the HCMO group exhibit the highest peak VO_2_ and peak C(a-v)O_2_ among groups, while peak CI, although not significantly different, is surprisingly lower than in ATTR-CA and HFpEF. Peak SVi values are similar across groups, despite the higher baseline SVi in HCMO.

Thus, functional limitation in HCMO may be primarily driven by LVOTO, with the blunted VO_2_ response further constrained by chronotropic incompetence, likely secondary to β-blocker use. In line with this observation, a previous study has reported that chronotropic impairment in HCM is attenuated after adjusting for β-blocker therapy [18]. Notably, these observations underscore the need for careful β-blocker titration to achieve effective LVOTO reduction without excessively compromising HR increase. Referring to the HFpEF group, the cardiac response is the most efficient among the cohort, with higher peak CI and peak SVi, while filling pressures remain lower.

However, peak C(a-v)O_2_ is the lowest overall, limiting peak VO_2_ to below the prognostic threshold of 14 mL/kg/min. This is further supported by a significantly higher CO/VO_2_ slope, suggesting that exercise performance may be primarily constrained by peripheral oxygen extraction inefficiency. Although our HFpEF group represents a heterogeneous cohort comprising both hypertensive heart disease and non-obstructive HCM patients, our findings align with existing evidence [26]. In this context, the CPET-ESE approach utilizes the inverse Fick equation to enable a non-invasive assessment of C(a-v)O_2_, which may offer additional clinical utility across the broader HFpEF spectrum [14].

In the ATTR-CA group, a pattern of global functional impairment is observed. While peak C(a-v)O_2_ is reduced, diastolic function, as assessed by E/e′, remains unchanged from rest to peak. Peak systolic indices are within normal limits, yet lower than in HFpEF. Notably, chronotropic reserve is the highest among groups.

Functional limitation therefore may result from combined central and peripheral mechanisms, with CO primarily sustained by chronotropic response, a finding consistent with previous reports [27]. These findings may potentially underscore the need for cautious β-blocker use in patients with cardiac amyloidosis.

Potential insights may also be gleaned from assessing right heart function. In patients with HCMO, the ΔTAPSE/PASP from rest to peak is positive, whereas it is negative in ATTR-CA and HFpEF, suggesting impaired right ventricular–pulmonary arterial coupling in the latter groups. Regarding HCMO, this finding is supported by improvement in diastolic function during exercise, as testified by baseline and peak E/e′ values.

Furthermore, despite higher baseline values, peak PASP remains relatively lower in HCMO, whereas the rise is greater in HFpEF and ATTR-CA.

In the HFpEF group, the observation of lower peak E/e′ values argues against RV–AP uncoupling being driven by elevated left ventricular filling pressures, indicating that this phenomenon may primarily reflect reduced pulmonary vascular compliance.

A similar pattern is observed in ATTR-CA, where, alongside reduced pulmonary vascular compliance, right ventricular function is also less efficient compared with HFpEF. Nevertheless, these findings remain exploratory. In contrast to our results, emerging evidence suggests that exercise-induced pulmonary hypertension (EiPAH) may play a key role in predicting adverse clinical outcomes in HCM [15].

### 5.3. Prediction of Functional Parameters

Multivariable analyses shows that several CPET-derived functional parameters can be predicted by integrating clinical, laboratory, and echocardiographic data, including stress measurements.

These associations might be physiologically plausible: peak VO_2_ is related to baseline TAPSE and peak CI, highlighting the role of global cardiocirculatory function in exercise capacity, while VE/VCO_2_ slope correlated with peak CI and LAVI, linking both systolic and diastolic dysfunction to ventilatory inefficiency.

Peak C(a-v)O_2_ results associated with baseline MR, peak CI, NT-proBNP and age, potentially reflecting the interaction between cardiac function and peripheral oxygen extraction, as well as the contribution of age-related sarcopenia.

Notably, E/e′ is not retained in the multivariable models, likely due to collinearity with other markers of pressure load and remodelling such as LAVI. Subgroup analyses yield similar trends but are not reported due to the limited sample size.

## 6. Conclusions

### 6.1. Strengths and Perspectives

The combined CPET-ESE approach offers a non-invasive, integrative assessment of cardiac, respiratory, and metabolic responses to exercise. By combining multiparametric variables such as VO_2_, VE/VCO_2_ slope and C(a-v)O_2_, it may provide a more comprehensive clinical characterisation across diagnostic, therapeutic, and prognostic domains by:

- Enhancing diagnostic accuracy by clarifying the underlying mechanism of exercise intolerance;
- Detecting latent LVOTO, a key prognostic and diagnostic hallmark in HCM that may be absent at rest but becomes evident during exertion;
- Guiding pharmacological management, allowing clinicians to tailor therapies such as β-blockers, diuretics, afterload-reducing agents, etc., after honing in on the predominant abnormality revealed during exercise;
- Assisting in the selection of invasive therapies, informing decisions regarding candidacy for septal reduction interventions, guiding pacing strategies, and supporting the design of personalised exercise rehabilitation programs;
- Enabling longitudinal monitoring of therapeutic response, which is particularly valuable in chronic settings.

In addiction, the development of predictive models linking clinical, laboratory and resting echocardiographic variables may expand the translational value of this approach. Indeed, these models can approximate key functional outcomes even in the absence of full CPET performance. This might be especially advantageous in resource-limited settings, in patients unable to perform maximal exercise testing, or in longitudinal follow-up when repeated CPET-ESE is impractical.

### 6.2. Limitations

As a diagnostic tool, CPET-ESE entails methodological constraints. Performance is highly effort-dependent and influenced by deconditioning, training status, and concurrent medications. From a technical perspective, the procedure requires specialised equipment and expertise for rapid multi-window image acquisition. Notably, the semi-supine position, necessary for image quality, can alter exercise biomechanics and shift the AT toward peak effort. Furthermore, respiratory and metabolic measurements are sensitive to calibration errors and day-to-day physiological variability. Regarding the study setting, recruitment from a single tertiary centre introduces inherent selection bias, as the cohort may reflect local referral patterns, operator expertise, and institutional workflows. Consequently, the inclusion of more selected, diagnostically complex, or symptomatic patients limits external validity. This cohort should therefore be regarded as hypothesis-generating rather than fully representative of hypertrophic phenotypes. In this regard, we acknowledge the current heterogeneity of the HFpEF group, which encompasses both hypertensive heart disease and non-obstructive HCM. Combined with the current sample size, this precludes subgroup comparisons or group-specific regression analyses at this stage. However, such detailed evaluations will be implemented as further recruitment expands the study population.

## Figures and Tables

**Table 1:** Parameters assessed by CPET-ESE across exercise stages.

Category	Parameter	Measurement Timepoint
**Clinical Hemodynamic Parameters**	SBP	Every 2 min
	DBP	Every 2 min
	HR	Continuous
**Exercise Stress Echocardiography**	LVEF	R; L–L; AT; P; Rec; Δ(P–R)
	SVi	R; L–L; AT; P; Rec; Δ(P–R)
	CI	R; L–L; AT; P; Rec; Δ(P–R)
	GLS	R; P
	E/e′ avg	R; L–L; AT; P; Rec; Δ(P–R)
	MR grade	R; L–L; AT; P
	PASP	R; L–L; AT; P; Rec; Δ(P–R)
	TAPSE	R; L–L; AT; P; Rec; Δ(P–R)
	TAPSE/PASP	R; P; Δ(P–R)
**Cardiopulmonary Exercise Test**	VO_2_	R; L–L; AT; P; Δ(P–R)
	VE	Continuous
	VT	Continuous
	RR	Continuous
	VE/VO_2_	Continuous
	VE/VCO_2_ slope	Entire exercise
	PETO_2_	Continuous
	PETCO_2_	Continuous
	O_2_ pulse	R; P; Δ(P–R)
	VO_2_/WR	Entire exercise
	VO_2_/HR	R; AT; P
	RER	P
	Chronotropic reserve	Derived (R; P)
	Ventilatory reserve	Derived (R; P)
**Integrated Cardiometabolic Parameters**	C(a-v)O_2_ difference	R; P; Δ(P–R)
	CO/VO_2_ slope	Entire exercise
	ΔSV/ΔVO_2_	Δ-derived (P–R)

This table summarises hemodynamic, echocardiographic, ventilatory, metabolic, and integrated cardiometabolic parameters. Timepoints: **R** = rest; **L–L** = low load; **AT** = anaerobic threshold; **P** = peak; **Rec** = early recovery; Δ**(P–R)** = change from peak to rest. Abbreviations: **C(a-v)O_2_** = arteriovenous oxygen difference; **CI** = cardiac index; **CO** = cardiac output; **DBP** = diastolic blood pressure; **E/e′ avg** = average ratio of early mitral inflow (E) to mitral annular e′ velocity; **GLS** = global longitudinal strain; **HR** = heart rate; **LVEF** = left-ventricular ejection fraction; **MR** = mitral regurgitation; **O_2_ pulse** = oxygen pulse; **PASP** = pulmonary artery systolic pressure; **PETCO_2_** = end-tidal CO_2_ pressure; **PETO_2_** = end-tidal O_2_ pressure; **RER** = respiratory exchange ratio; **RR** = respiratory rate; **SBP** = systolic blood pressure; **SVi** = stroke volume index; **TAPSE** = tricuspid annular plane systolic excursion; **VE** = minute ventilation; **VCO_2_** = carbon dioxide production; **VO_2_** = oxygen uptake; **WR** = work rate.

**Table 2:** Clinical parameters, risk factors and biomarkers.

	General Population (*n* = 43)	ATTR-CA (*n* = 15)	HFpEF (*n* = 9)	HCMO (*n* = 19)	*p* Value < 0.05 *
Age, (years)	68 ± 10	73 ± 9	74 ± 7	62 ± 9	0.001 *
BSA, (m^2^)	2.0 ± 0.3	2.0 ± 0.2	2.0 ± 0.2	2.0 ± 0.3	0.92
NYHA II class, *n* (%)	32(74%)	9(60%)	5(56%)	18(95%)	0.024 *
Hypertension, *n* (%)	34(79%)	9(60%)	8(89%)	17(90%)	0.08
Diabetes, *n* (%)	5(12%)	2(13%)	2(22%)	5(26%)	0.41
Smoker, *n* (%)	17(40%)	7(47%)	2(22%)	8(42%)	0.59
AF, *n* (%)	15(35%)	6(40%)	4(44%)	5(26%)	0.33
CAD, *n* (%)	10(23%)	6(40%)	3(33%)	1(5%)	0.037 *
logTnT	1261 (1055–1433)	1582(1359-1655)	1063(1000–1202)	1128(1023–1246)	<0.001 *
NTproBNP, (pg/mL)	933(459–1573)	1007(650–1697)	793(132–1714)	693(447–1652)	0.62
EGFR, (mL/min/1.73 m^2^)	75 ± 17	69 ± 20	71 ± 8	79 ± 15	0.23
β-blocker, *n* (%)	31(72%)	8(53%)	6(67%)	17(90%)	0.061
ACEi/ARB/ARNI, *n* (%)	28(65%)	10(67%)	5(56%)	13(68%)	0.79
MRA, *n* (%)	11(26%)	6(40%)	1(11%)	4(21%)	0.24
SGLT2i, *n* (%)	4(9%)	2(13%)	1(11%)	1(5%)	0.71
Loop diuretic, *n* (%)	3(7%)	3(20%)	0(0%)	0(0%)	0.063

**ACEi** = Angiotensin-Converting Enzyme inhibitors; **AF** = atrial fibrillation; **ARB** = Angiotensin II Receptor Blockers; **ARNI** = Angiotensin Receptor–Neprilysin Inhibitors; **BSA** = body surface area; **CAD** = coronary artery disease; **EGFR** = estimated glomerular filtration rate; **MRA** = Mineralocorticoid Receptor Antagonists; **NTproBNP** = N-terminal pro-B-type natriuretic peptide; **NYHA** = New York Heart Association; **SGLT2i** = Sodium–Glucose Cotransporter-2 inhibitors; **TnT** = troponin T. * = p<0.05 is considered statistically significant.

**Table 3:** Parameters measured at basal echocardiography.

	General Population (*n* = 43)	ATTR-CA (*n* = 15)	HFpEF (*n* = 9)	HCMO (*n* = 19)	*p* Value < 0.05 *
LVMi, (g/m^2^)	158 ± 38	162 ± 36	136 ± 27	163 ± 41	0.26
IVSd, (mm)	17.0 ± 2.9	16.7 ± 1.8	14.9 ± 2.3	18.2 ± 3.3	0.025 *
PWd, (mm)	13.1 ± 2.4	14.5 ± 2.0	11.4 ± 1.1	12.5 ± 2.6	0.008 *
EDD, (mm)	48 ± 5	48 ± 6	50 ± 5	48 ± 4	0.52
EDV, (mL)	121 ± 33	107 ± 31	121 ± 32	132 ± 33	0.09
EF, (%)	59 ± 6	56 ± 7	59 ± 5	61 ± 5	0.022 *
E/e′ avg	13.7 ± 4.7	14.7 ± 5.4	10.1 ± 3.1	14.6 ± 4.0	0.030 *
TAPSE, (mm)	21 ± 4	20 ± 4	20 ± 3	23 ± 3	0.051
PASP, (mmHg)	34 ± 9	33 ± 10	33 ± 7	34 ± 8	0.93
LAVI, (mL/m^2^)	40 ± 10	41 ± 11	40 ± 12	40 ± 8	0.91
Vmax Ao, (cm/s)	192 ± 93	150 ± 57	151 ± 52	247 ± 106	0.002 *
≥moderate MR, *n* (%)	8(19%)	2(13%)	0(0%)	6(32%)	0.024 *
≥moderate TR, *n* (%)	3(7%)	1(7%)	2(22%)	0(0%)	0.046 *

**EDD** = end-diastolic diameter; **EDV** = end-diastolic volume; **EF** = ejection fraction; **E/e′ avg** = average E-wave to e′-wave ratio; **IVSd** = interventricular septal thickness in diastole; **LAVI** = left atrial volume index; **LVMi** = left ventricular mass index; **MR** = mitral regurgitation; **PASP** = systolic pulmonary artery pressure; **PWd** = posterior wall thickness in diastole; **TAPSE** = tricuspid annular plane systolic excursion; **TR** = tricuspid regurgitation; **Vmax Ao** = maximum aortic velocity. * = p<0.05 is considered statistically significant.

**Table 4:** Parameters measured by CPET-ESE at baseline and at peak exercise.

	General Population (*n* = 43)	ATTR-CA (*n* = 15)	HFpEF (*n* = 9)	HCMO (*n* = 19)	*p* Value < 0.05 *
basal SBP, (mmHg)	131 ± 19	130 ± 20	130 ± 23	133 ± 16	0.84
basal DBP, (mmHg)	74 ± 10	71 ± 11	72 ± 12	76 ± 8	0.30
basal HR, (bpm)	64 ± 12	66 ± 12	68 ± 11	60 ± 12	0.24
basal CI, (L/min/m^2^)	2.3 ± 0.6	2.2 ± 0.5	2.3 ± 0.5	2.4 ± 0.7	0.44
basal SVi, (mL/m^2^)	37 ± 9	35 ± 10	36 ± 5	40 ± 9	0.15
basal E/e′ avg	12.7 ± 5.0	12.5 ± 5.2	9.1 ± 4.1	14.6 ± 4.2	0.017 *
basal TAPSE, (mm)	22 ± 3	20 ± 3	22 ± 4	23 ± 3	0.052
basal PASP, (mmHg)	32 ± 8	29 ± 5	31 ± 8	34 ± 9	0.14
basal TAPSE/PASP, (mm/mmHg)	0.72 ± 0.21	0.73 ± 0.21	0.78 ± 0.27	0.70 ± 0.18	0.69
basal VO_2_, (mL/min/Kg)	3.85 ± 1.57	3.04 ± 1.36	2.90 ± 0.63	4.92 ± 1.40	<0.001 *
basal C(a-v)O_2_, (mL/100 mL)	7.24 ± 3.07	6.20 ± 3.39	5.50 ± 1.36	8.88 ± 2.64	0.004 *
peak SBP, (mmHg)	184 ± 27	180 ± 27	176 ± 32	191 ± 24	0.27
peak DBP, (mmHg)	91 ± 15	85 ± 15	89 ± 17	95 ± 12	0.15
peak HR, (bpm)	106 ± 19	114 ± 22	108 ± 19	98 ± 14	0.055
peak CI, (L/min/m^2^)	5.1 ± 1.4	5.1 ± 1.7	5.5 ± 1.5	4.8 ± 1.1	0.56
peak SVi, (mL/m^2^)	48 ± 12	44 ± 13	50 ± 8	49 ± 12	0.37
peak E/e’ avg	12.0 ± 4.6	12.3 ± 4.0	8.6 ± 3.8	13.4 ± 4.8	0.033 *
peak PASP, (mmHg)	45 ± 18	50 ± 14	48 ± 14	42 ± 21	0.44
peak ≥ moderate MR, *n* (%)	15 (37%)	3 (21%)	0 (0%)	12 (63%)	0.003 *
peak TAPSE, (mm)	27 ± 5	26 ± 5	28 ± 6	26 ± 4	0.38
peak TAPSE/PASP, (mm/mmHg)	0.69 ± 0.35	0.55 ± 0.20	0.68 ± 0.34	0.79 ± 0.41	0.17
peak VO_2_, (mL/min/Kg)	14.92 ± 3.98	13.55 ± 4.73	13.27 ± 3.34	16.78 ± 2.84	0.020 *
peak C(a-v)O_2_, (mL/100 mL)	12.77 ± 3.37	11.48 ± 3.07	10.46 ± 1.97	14.88 ± 2.94	<0.001 *
ΔTAPSE, (mm)	5 ± 3	6 ± 3	6 ± 4	3 ± 2	0.047 *
ΔTAPSE/PASP, (mm/mmHg)	−0.03 ± 0.31	−0.18 ± 0.16	−0.10 ± 0.16	0.10 ± 0.40	0.045 *
AT reached, *n* (%)	20 (47%)	1 (7%)	2 (22%)	17 (90%)	<0.001 *
Chronotropic reserve, (%)	51 ± 23	62 ± 22	57 ± 32	39 ± 12	0.009 *
CO/VO_2_ slope	6.07 ± 2.53	6.77 ± 2.57	7.25 ± 3.02	4.96 ± 1.82	0.029 *
VE/VCO_2_ slope	28.3 ± 5.1	29.2 ± 7.1	27.9 ± 3.6	27.7 ± 3.7	0.70

**AT** = anaerobic threshold; **CI** = cardiac index; **CO/VO_2_ slope** = slope of the relationship between cardiac output and oxygen consumption; **C(a-v)O_2_** = arteriovenous oxygen content difference; **DBP** = diastolic blood pressure; **E/e′ avg** = average E-wave to e′-wave ratio; **HR** = heart rate; **MR** = mitral regurgitation; **PASP** = systolic pulmonary artery pressure; **SVi** = stroke volume index; **SBP** = systolic blood pressure; **TAPSE** = tricuspid annular plane systolic excursion; **VE/VCO_2_ slope** = slope of the relationship between minute ventilation and carbon dioxide production; **VO_2_** = oxygen consumption. * = p<0.05 is considered statistically significant.

## Data Availability

The data underlying this article will be shared on reasonable request to the corresponding author.

## Supplementary Materials

The following supporting information can be downloaded at: https://www.mdpi.com/article/10.3390/jcm15093470/s1.

## Abbreviations

The following abbreviations are used in this manuscript:

AF	Atrial Fibrillation;
ATTR-CA	Transthyretin Cardiac Amyloidosis;
AUC	Area Under the Curve;
BMI	Body Mass Index;
BSA	Body Surface Area;
CI	Cardiac Index;
COPD	Chronic Obstructive Pulmonary Disease;
CPET	Cardiopulmonary Exercise Testing;
CPET-ESE	Combined Cardiopulmonary Exercise Test and Exercise Stress Echocardiography;
DP	Double Product;
E/e′	Ratio of Early Mitral Inflow to Mitral Annular e′ Velocity;
EF	Ejection Fraction;
EiPAH	Exercise-induced Pulmonary Hypertension;
eGFR	Estimated Glomerular Filtration Rate;
ESE	Exercise Stress Echocardiography;
FEV1	Forced Expiratory Volume in 1 s;
GLS	Global Longitudinal Strain;
HCM	Hypertrophic Cardiomyopathy;
HCMO	Hypertrophic Cardiomyopathy with Obstruction;
HFpEF	Heart Failure with Preserved Ejection Fraction;
HFrEF	Heart Failure with Reduced Ejection Fraction;
HR	Heart Rate;
IVSd	Interventricular Septal Thickness in Diastole;
LAVI	Left Atrial Volume Index;
LogTnT	Log-transformed cardiac Troponin T;
LVEDD	Left Ventricular End-Diastolic Diameter;
LVEDV	Left Ventricular End-Diastolic Volume;
LVH	Left Ventricular Hypertrophy;
LVMi	Left Ventricular Mass Index;
LVOT	Left Ventricular Outflow Tract;
LVOTO	Left Ventricular Outflow Tract Obstruction;
mMRC	modified Medical Research Council dyspnea scale;
MR	Mitral Regurgitation;
MRA	Mineralocorticoid Receptor Antagonist;
NT-proBNP	N-terminal pro–B-type Natriuretic Peptide;
PASP	Pulmonary Artery Systolic Pressure;
PETCO_2_	End-Tidal Carbon Dioxide Pressure;
PWd	Posterior Wall Thickness in Diastole;
RER	Respiratory Exchange Ratio;
SAM	Systolic Anterior Movement;
SGLT2i	Sodium/Glucose Cotransporter-2 Inhibitor;
SV	Stroke Volume;
SVi	Stroke Volume Index;
TAPSE	Tricuspid Annular Plane Systolic Excursion;
VE	Minute Ventilation;
VE/VCO_2_	Ventilatory Equivalent for CO_2_;
VIF	Variance inflation Factor;
VO_2_	Oxygen Uptake
