# Supplementary material for: Functional Assessment of Hypertrophic Phenotype Cardiomyopathies Using Combined Cardiopulmonary Exercise Testing and Echocardiography: A Pilot Single-Centre Study
Source: J Clin Med. 2026 May 1;15(9):3470. doi: 10.3390/jcm15093470 (PMC13163232; doi:10.3390/jcm15093470)
Supplement: Supplementary file 1 [file jcm-15-03470-s001.zip › jcm-4246940-supplementary.pdf]

Parameters examined by CPET-SE

Cardiac parameters

The following parameters are utilized to describe the dynamic myocardial and haemodynamic response during incremental exercise:

- Stroke volume index (Svi), cardiac index (CI), Left Ventricular Ejection Fraction (EF) and Global Longitudinal Strain (GLS), including their specific variations across the different stages of exercise;
- E/e′ average ratio: serving as a non-invasive surrogate of left-ventricular filling pressures;
- TAPSE and PASP: reflecting right-ventricular systolic performance and pulmonary vascular load during exertion;
- TAPSE/PASP ratio: an established marker of right ventricle-pulmonary artery coupling, capturing the ability of the right ventricle to adapt to rising afterload;
- Exercise-induced MR: assessed semi-quantitatively across all stages (rest, low load, anaerobic threshold, and peak), with particular focus on its relevance in HFpEF and obstructive HCM;

2 Ventilatory parameters

Ventilatory efficiency and breathing patterns are quantified using continuous breath-by-breath data:

- Minute ventilation (VE), tidal volume (VT), and respiratory rate (RR);
- End-tidal CO_2 pressure ($PETCO_2$) and End-tidal O_2 pressure ($PETO_2$), providing insights into gas exchange efficiency;
- VE/VCO_2 and VE/VO_2 ratios, and the VE/VCO_2 slope: established markers of ventilatory efficiency and chemoreceptor drive;
- Ventilatory reserve: derived from the ratio between peak VE and the estimated maximal voluntary ventilation, used to identify ventilatory limitation.

2 Metabolic parameters

Metabolic performance and oxygen kinetics are evaluated using derived indices:

- VO_2 across workloads, O_2 pulse (VO_2/HR), and arteriovenous oxygen difference [$C(a–v)O_2$]: describing the balance between cardiac output delivery and peripheral oxygen extraction;
- CO/VO_2 slope: a composite metric of circulatory efficiency that integrates cardiac output and metabolic demand;
- VO_2/WR slope: reflecting the proportionality between metabolic performance and external workload;

- Chronotropic reserve: derived from the relationship between predicted and attained heart rate (HR), providing information on cardiac autonomic function and β-adrenergic responsiveness.

Mechanisms of exercise limitation are classified as cardiovascular, ventilatory, ventilation–perfusion mismatch, or metabolic using predefined thresholds (e.g., peak VO_2 <14 mL/kg/min, VE/VCO_2 slope >34, peak $PETCO_2$ <33 mmHg, or peak $C(a–v)O_2$ <13 mL/dL). Within the cardiovascular domain, further subclassification is performed based on chronotropic incompetence, reduced cardiac output reserve, and an E/e′ ratio >14 during exertion.
